# Influence of Livelihood Strategies on Local People Perception Toward the Benefits and Cost of Community‐Based Natural Resource Management: A Case of Burunge Wildlife Management Area, Tanzania

**DOI:** 10.1002/ece3.73130

**Published:** 2026-02-19

**Authors:** Juma J. Kegamba, Agustino S. Melembuki, Jackline J. Kyaruzi

**Affiliations:** ^1^ Department of Wildlife Management College of African Wildlife Management (CAWM) Moshi Kilimanjaro Tanzania; ^2^ African Wildlife Foundation (AWF) Morogoro Tanzania

**Keywords:** agro‐pastoralist, Burunge Wildlife Management Area (BWMA), community‐based natural resource management (CBNRM), minority groups, wildlife management areas (WMAs)

## Abstract

Involving local people in the sustainable conservation of biodiversity is a global issue that requires urgent attention for better conservation outcomes. Understanding the views of those living next to the protected areas such as community‐based natural resource management (CBNRM) is critical to decision making in achieving the desired conservation goals. This study used a semi‐structured questionnaire approach (self‐administered) in a novel investigation of the perception toward the benefits and costs, and the level of support by local people with differing livelihood strategies to Burunge Wildlife Management Area (BWMA). In total, 302 respondents from the two purposively selected villages out of 10 which form the BWMA were surveyed: Minjingu (147) and Vilima Vitatu (155). We found that the large number of the respondents (73%, *n* = 170) from the majority agro‐pastoralist group showed a negative perception toward the benefits received from the BWMA. Their negative perception was associated with the cost they experience from BWMA and the long‐term need of the grazing land from the area. On the contrary, respondents from minority groups (i.e., fish mongers, business, and weaving) were significantly positive toward the benefits of the BWMA and its existence, as their livelihood strategies are highly dependent on it. Furthermore, the level of support for the existence of the BWMA was different between the two surveyed villages, which might be due to the level of conflicts they have with its operations. Investigations of local perceptions toward natural resources management should not be limited to the groups in a society that have the majority livelihood strategy, but should also consider those whose livelihood strategies are in the minority and their voices largely ignored.

## Graphical abstract

This study examined local perceptions of Burunge Wildlife Management Area (BWMA) in Tanzania using semi‐structured questionnaires with 302 respondents from two villages. Findings revealed that the majority agro‐pastoralist group held negative views of BWMA due to grazing restrictions and associated costs, while minority groups such as fishmongers, businesspeople, and weavers expressed strong support because their livelihoods depend on it. The results highlight the importance of considering both majority and minority livelihood groups in community‐based natural resource management (CBNRM) to achieve equitable and sustainable conservation outcomes.

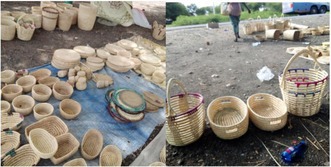

## 1 Introduction

Community‐based natural resource management (CBNRM) in sub‐Saharan Africa emerged as a decentralized alternative to an earlier centralized conservation mode commonly referred to as “fortress” or “fence and fine” (Neumann 1998; Kegamba et al. 2022). The former model emphasized the enforcement of strict rules governing the use of natural resources. Consequently, local communities were often excluded from decision‐making processes and experienced restricted access to natural resources (Songorwa et al. 2000; Nelson and Agrawal 2008; Ribot et al. 2010). The model prioritized protection and enforcement, which frequently led to local conflicts and resulted in a lack of support from communities surrounding protected areas (Igoe 2002). Therefore, decentralized CBNRM was thought to be a new liberation to integrate conservation goals, needs, and the development of local communities, by emphasizing their active participation and equitable sharing of benefits derived from conservation (Igoe and Croucher 2007). As a result, several CBNRM projects based on the use of wildlife resources were established in sub‐Saharan Africa in the 1990s and 2000s. Examples include the Communal Areas Management Program for Indigenous Resources (CAMPFIRE) in Zimbabwe (Mberengwa 2000), Community Conservancies in Namibia, the Administrative Management and Design for Game Management Areas (ADMADE) in Zambia (Mwenya et al. 1990; DeGeorges 1992), and the community Wildlife Management Areas (WMAs) in Tanzania (URT 2007). However, there have been varying opinions on whether this neoliberal ideology has managed to turn things around, especially in social outcomes and securing local support (Galvin et al. 2018). In connection to these outcomes is the question of whether marginalized groups are being heard on issues customarily perceived as within the majority domain (WWF‐USAID 2014a, 2014b). Evidence is vital to judge how various groups with different livelihood strategies bordering a CBNRM project such as the WMAs of Tanzania perceive the benefits from and costs of a conservation area and how they support the continued existence of these conserved areas.

The Zimbabwean CAMPFIRE project (Martin 1986) has been highlighted in several studies as a model example of CBNRM for its success in creating local employment and infrastructure development (Mutandwa and Gadzirayi 2007; Tchakatumba et al. 2019; Shereni and Saarinen 2021; Phiri et al. 2024). However, many of these successes in providing local benefits and therefore securing community support were associated with the investments and external funding of large donors (Balint and Mashinya 2008; Shereni and Saarinen 2021). The negative local perception of CAMPFIRE projects began to be expressed after the Zimbabwean economic crisis that led to the withdrawal of external funding for these projects (Balint and Mashinya 2008; Dzvimbo et al. 2018; Tchakatumba et al. 2019; Phiri et al. 2024). In addition, the local people next to some CAMPFIRE projects perceived that there was an increase in human wildlife conflict (HWC), which continued to negatively impact community livelihood (Mutandwa and Gadzirayi 2007; Gandiwa et al. 2013; Matanzima and Marowa 2022). Furthermore, it was alleged that some CAMPFIRE projects neglected to include the views of minority groups dealing with other livelihood practices, such as business and artisanal weaving (Jani et al. 2024).

A WMA is an area of communal land that has been designated by the member villages as habitat for wildlife (Wildlife Conservation Act No. 5 of 2009, revised 2022). The establishment of WMAs aimed to hand over the management of wildlife to local communities, in order to create local motivation for conservation (Benjaminsen et al. 2013). The key underlying assumptions of the WMA concept are to provide local communities with economic benefits by directly involving them in the management of wildlife resources. As a result, the WMAs were designed to promote both the long‐term conservation of wildlife and local economic development. The intention was that communities would develop an interest in the conservation of natural resources due to the direct benefits received from conservation. These concepts and objectives of the WMAs were maintained in the revised 2007 wildlife policy (URT 2007).

In Tanzania, the establishment of a Wildlife Management Area (WMA) follows a legally defined process that requires the voluntary participation and consent of member villages (GN No. 381 of 2018, Sections 9–11). Villages intending to form a WMA must formally set aside portions of their communal village land for wildlife conservation through village assembly approval and form an Authorized Association (AA) to manage the designated area on their behalf. Once these requirements are met, the proposed WMA is registered by the central government, and management authority over wildlife use and benefit‐sharing is transferred to the AA under national wildlife legislation. In principle, this process is intended to ensure local ownership, participation, and informed decision‐making in conservation initiatives. Currently, there are 38 WMAs at different stages of development (out of these, 22 are fully registered and operational) covering approximately 13% of the land in Tanzania, and all are located in the buffer zones of the core protected areas (i.e., National Parks and Game Reserves) (Kicheleri et al. 2018). However, the socioeconomic benefits and costs associated with WMAs trigger varying opinions toward WMAs among those communities highly affected by conservation (Homewood et al. 2022). People whose livelihood strategies place them in a majority and therefore favored group in the WMAs can gain advantages of being heard, and influence the outcomes more than those marginalized due to gender, ethnicity, and political, cultural, or economic systems (Homewood et al. 2022).

The Burunge Wildlife Management Area (BWMA), established in 2003, is one of the leading revenue‐generating WMAs in Tanzania (Veit 2019). Like many WMAs in Tanzania, BWMA has experienced conflicts between the central government and district authorities over the allocation and use of benefits generated from it (Igoe and Croucher 2007; Bluwstein et al. 2016; Kicheleri et al. 2018, 2021). The central government has been alleged to interfere with BWMA operations by making critical decisions, including utilization of natural resources (Nelson and Agrawal 2008; Benjaminsen et al. 2013). These conflicts led to one participating village (Minjingu) filing a case in 2014 at the High Court of Tanzania to withdraw from the association (Bluwstein et al. 2016; Kicheleri et al. 2018, 2021). The decision to file such a vital case was reached at the village meeting and was endorsed by majority of the villagers, as Kicheleri et al. (2018) and Kicheleri et al. (2021) observed in detail. The question was whether the voices of minority groups with different livelihood strategies were considered before filing the case to the court. The court ruled in favor of Minjingu village, but after several negotiations with the central government outside the court system (Kicheleri et al. 2021), Minjigu village decided to stay in the association. These various conflicts between the central government and the villages forming the WMAs in Tanzania prompted Kicheleri et al. (2021) to critically describe this CBNRM project in Tanzania, as being designed by the central government for the accumulation of more revenues by dispossessing local populations.

A key constraint in securing community support has been a mismatch of interest between conservation authorities and local communities, especially on the impact of conservation on their livelihood strategies. The situation is further complicated for BWMA due to differences in terms of livelihood activities conducted by local people living near the reserve (Moyo et al. 2017). Adding to these complications is the direct association and connection between most of those local livelihood strategies near BWMA and the reserved land. The communities around BWMA include the majority agro‐pastoralists who focus on cropping and raising livestock but depend on the reserved areas to supplement fodder (Moyo et al. 2017). In addition to this, their crops and livestock are destroyed by marauding animals from the same BWMA and nearby protected areas (Tarimo et al. 2021; Hariohay et al. 2024). In addition, there are minority groups including fishers and fish mongers who depend on reserved Lake Burunge and nearby Lake Manyara as a source to earn a living, while the small business people depend on the increasing number of tourists in the areas, which increase multiplier effect for their economic gains. Similarly, the artisanal heritage minority depend on weaving materials that are mostly protected in the BWMA to make their products and gain income. These differences in culture, livelihood strategies, and the local history are likely to have profound effects on local perceptions of and support for the continued existence of nearby protected area (Kegamba et al. 2023).

While several studies have examined community perceptions of WMAs in Tanzania, most have focused on dominant livelihood groups, particularly agro‐pastoralists, who often experience direct costs such as land‐use restrictions and human–wildlife conflict (Tarimo et al. 2021; Hariohay et al. 2024; Kegamba, Sangha, Wurm, Meitamei, et al. 2024). As a result, much less is known about how minority livelihood groups such as fishers, small business owners, and artisanal weavers perceive the benefits, costs, and overall value of WMAs. This lack of attention limits our understanding of whose voices are represented in conservation decision‐making and whether WMAs equitably support all affected community members. This study addresses this knowledge gap by investigating the perceptions of and the level of support for BWMA, by local populations with differing livelihood strategies (the majority agro‐pastoralists and the minority fishers, fish mongers, small business owners, and artisanal weavers). These groups of people are also affected by the conservation of BWMA. Specifically, we assessed the:

- Local perception of benefits received from BWMA.
- Local perception of costs to their livelihood activities from BWMA, and
- Level of local support for the continued existence of BWMA in their area.

## 2 Methodology

### 2.1 Study Area Description

**FIGURE 1:**
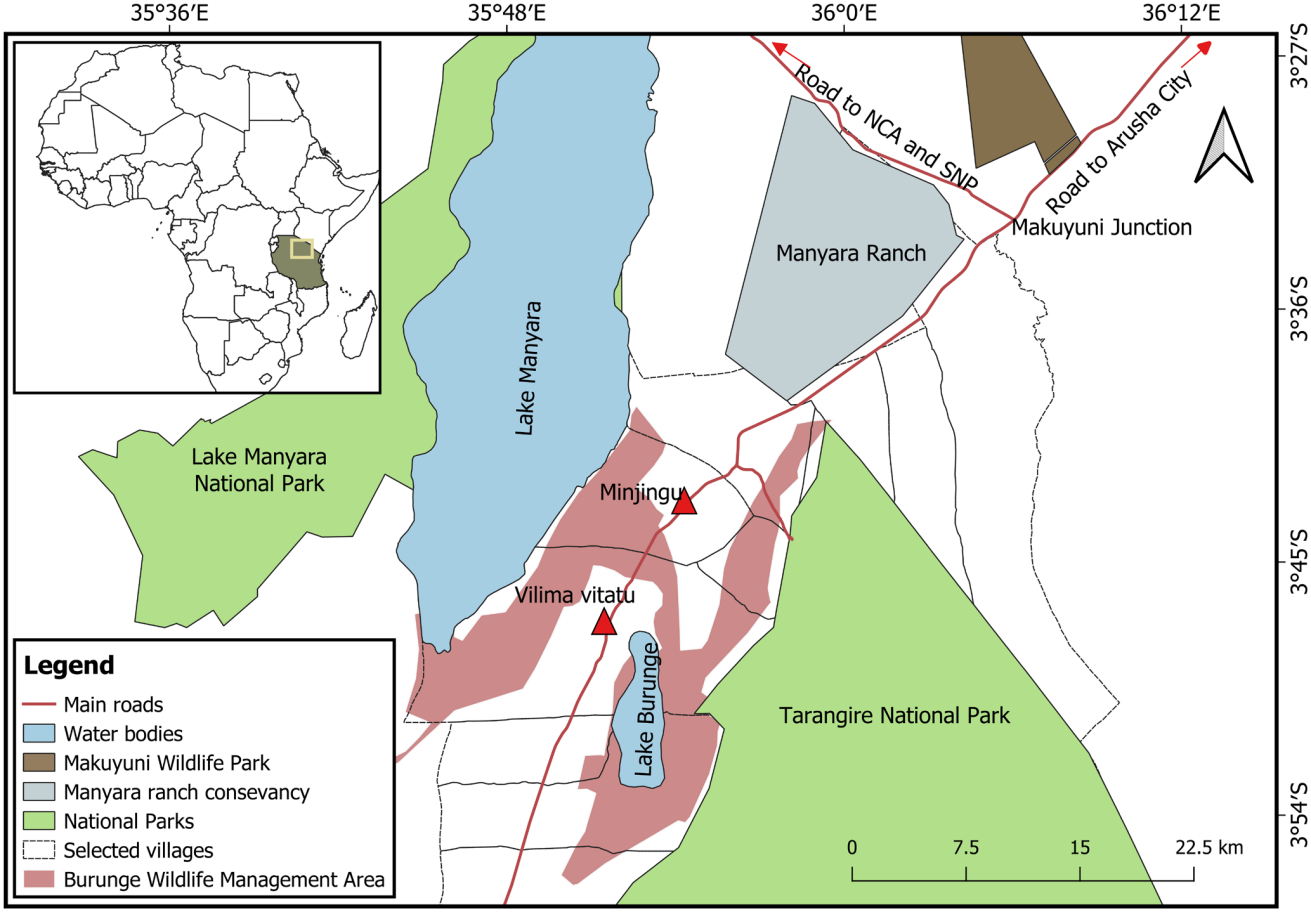
BWMA—central magenta H shape—with surveyed villages and nearby protected areas (inset Tanzania country location) (Created by authors using QGIS 3.28.2); NCA‐ Ngorongoro Conservation Area, SNP‐ Serengeti National Park.

This study was conducted in two villages: Minjingu and Vilima Vitatu, located in the Babati District, Manyara Region, Tanzania, East Africa. The two villages are among 10 forming the BWMA, located in the northern part of Tanzania between 03°57′ S to 03°40′ S and 35°44′ E to 35°58′ E. The area derived its name from Lake Burunge, which it contains. Lake Burunge is a crucial wildlife habitat for birds and mammals that regularly visit the area for water and is among the tourist attractions in the BWMA (Figure 1). The lake is home to a variety of migratory water birds, including both greater and lesser flamingos ( *Phoenicopterus roseus* and *Phoeniconaias minor*, respectively), as well as several species of ducks and shorebirds (Nelson et al. 2006). Lake Burunge is connected to Lake Manyara, the largest lake in the area, by a seasonal river canal, and both are in the foothills of the eastern part of the rift valley escarpment. BWMA is located in an area that is rich in wildlife and is a bridge between the most highly visited protected areas within the northern tourist circuit of Tanzania (Okello and Yerian 2009; Kimario et al. 2020), and therefore it has great potential for tourism and related business investments.

BWMA covers an area of 617 km^2^ (Chebby et al. 2024) and forms a wildlife corridor between Tarangire (2600 km^2^) and Lake Manyara national parks (648.7 km^2^), Makuyuni Wildlife Park (47 km^2^), Lake Natron Game Controlled Area (3000 km^2^), and Ngorongoro Conservation Area (8292 km^2^). BWMA is an area of high conservational significance because it adjoins those adjacent protected areas and their rich wildlife species such as buffalos, elephants, wildebeest, zebras, and birds ranging from terrestrial and water birds.

BWMA is traversed by the Arusha–Babati main road, which connects it to nearby major conservation and administrative centers, including Ngorongoro Conservation Area, Serengeti National Park, Arusha City, Babati District, and Dodoma, the national capital. This road enhances accessibility to the area, supports tourism development, and facilitates local trade and business opportunities. However, it also poses challenges, including increased wildlife–vehicle collisions and easier human access that may exacerbate resource pressure and illegal activities within the BWMA (Njovu et al. 2019).

BWMA or in *Kiswahili* called *Jumuiya ya Hifadhi ya Wanyamapori Burunge (JUHIBU)*, is bordered by the Lake Manyara and Tarangire national parks (NE. and SW. respectively) with the newly established Makuyuni Wildlife Park and Manyara Ranch about 35 km to the east (Lyimo et al. 2024). BWMA itself comprises 10 villages: Mwada, Sangaiwe, Ngoley, Vilima Vitatu, Kakoi, Olasiti, Manyara, Magara, Maweni, and Minjingu, and the ubiquitous presence of the rift valley dominates local and tourist perspectives from all the villages (Kicheleri et al. 2018). The villages are inhabited by more than 30,258 ethnic peoples, mostly Mbugwe, Arusha, Maasai, Barbaig, Iraqw, Nyaturu, and Nyiramba (NBS 2022). In theory, BWMA is managed by its Authorized Association (AA), a board of elected representatives from participating villages. While communities are consulted during the establishment of WMAs, key legislative and regulatory powers such as approval of management plans, quotas, and investment permits remain centralized within government institutions, thereby limiting the effective autonomy of AA (Kicheleri et al. 2018).

Most of the villagers are agro‐pastoralists (Chebby et al. 2023) growing different types of crops such as maize, sorghum, finger millet, and beans, mainly for food, and rice, sunflower, onions, garlic, watermelon, sesame, and cotton as cash crops. However, they also keep livestock, mainly cattle, goats, and sheep (Moyo 2018). The Maasai, Arusha, and Barbaig peoples tend to keep larger numbers of livestock than others, such as Iraqw ethnic group (Moyo et al. 2016). In addition, there are smaller groups dealing with other livelihood activities such as small business (shops, restaurants, and guest houses) in the village centers, fishing in Lake Burunge and Lake Manyara, and fish mongers in the village and along the Arusha‐Babati road (Kicheleri et al. 2018). Furthermore, there is another smaller group with artisanal weaving activities, who collect materials from designated areas inside BWMA and produce woven items sold to tourists and travelers along the highway (Mmbaga et al. 2024).

### 2.2 Data Collection

#### 2.2.1 Sampling

This study used a mixed‐method research approach in obtaining both qualitative and quantitative data (Taherdoost 2022). Information on whether the local people feel they receive benefits or experience costs from the presence of the BWMA and whether they support its continued existence was collected from communities in two purposively selected villages (i.e., Vilima Vitatu, and Minjingu) through an individual questionnaire survey (self‐administered). For data collection, we adopted the cluster sampling techniques of Kijazi and Kant (2011), which included clustered samples from members of the entire group. First, we categorized communities binarily, based on their relationship with BWMA management, as those with known conflict (sample target) or those with no known conflict (neutral sample target). This categorization was done based on literature and accessibility. Therefore, Minjingu village was selected as one of the villages known to have a conflict with BWMA to the extent of filing the case to the court to withdraw from the association (URT 2016a, 2016b; Kicheleri et al. 2018, 2021), and Vilima Vitatu village was selected as a neutral village (i.e., not known to have any conflict), but very accessible through the main road.

Second, we also used purposive nonprobability sampling technique (Stratton 2023) to select “representative livelihood strategies” among many livelihood strategies within those two selected villages (Cervigni et al. 2015; Zafra‐Calvo et al. 2017). In this sampling method, the researcher uses their own knowledge and judgment to choose a sample that is considered representative of the target population (Stratton 2023). In line with the available literature (Hariohay 2015; Kicheleri et al. 2018, 2021; Chebby et al. 2024) and the discussion with the local authorities we eventually selected agro‐pastoralism as the predominant activity (hereafter, majority group) of the local area populations, with those fewer in number—fishers and mongers (fish sellers) grouped together, small business owners, and artisanal weavers—as minority livelihood activities. Furthermore, we used the main income generating activity to the household to isolate those owning multiple economic activities. Before data collection, we obtained a research permit from the Tanzanian Commission of Science and Technology (COSTECH) No. 2021‐476‐NA‐2021‐170 and an introductory letter from the Babati District Council.

#### 2.2.2 Questionnaire Surveys

We surveyed 302 respondents in the BWMA study area from the two selected villages: Minjingu (*n* = 147) and Vilima Vitatu (*n* = 155). For the agro‐pastoralists (majority group), we randomly selected households and conducted a quasi‐structured questionnaire (self‐administered), defining a household as a group of individuals living together, usually with a single person self‐identifying as the head. If the household head was unavailable, we selected the nearest household instead. For the artisanal weavers, fish mongers, and small business owners (minority groups), we used purposive sampling techniques to identify the respondent (with the help of local leaders) and hence self‐administered the questionnaire in their area of operation. We began administering the questionnaire by seeking verbal consent from respondents and informing them of their right to withdraw at any time. If they chose to withdraw, we assured them that all recorded information would be removed from the research records. We asked respondents the following questions about their perceptions concerning the BWMA:

- Do you feel you receive benefits from BWMA?
- Do you feel that you experience costs from BWMA that affect your main livelihood activity?
- Do you support the continued existence of BWMA?

The answers to these questions were binary (yes or no), and if the answer was yes, we asked a follow‐up question. For questions (i) and (ii), respondents were asked a follow‐up open‐ended question on the type of benefits they received and the costs they experienced, respectively, and for question (iii), respondents were asked to rank the level of support they have for the continued existence of the BWMA in their area (answers recorded on a Likert scale: (1) Strongly oppose; (2) Oppose; (3) Neither oppose nor support; (4) Support; (5) Strongly support).

The questionnaires were primarily administered in Kiswahili, Tanzania's national language, in which the lead author is fluent. When necessary, a translator facilitated discussions in the local language. The questionnaires were hand‐written, recorded, manually transcribed, and translated into English for analysis by the lead author.

### 2.3 Data Analysis

We used binomial generalized linear models (binomial GLM) to assess how demographic factors (livelihood strategy, village, gender, age, and education level) influenced a binary agreement response variable. The models aimed to answer the following research questions regarding community perspectives on the BWMA, specifically whether:

- they feel they receive benefits from BWMA;
- they feel they experience costs from BWMA in their livelihood activity;
- they support the continued existence of BWMA in their area

We used AIC to select the best parsimonious model(s). We performed model validation using the DHARMa R package (Hartig 2020). We checked model residuals to assess collinearity and found no collinearity between predictors. We then used the ggplot2 R package (Wickham et al. 2016) for visualizations, and plots were merged using the patchwork R‐package (Pedersen 2024). All statistical analysis were performed using R version 3.6.2 (R Core Team 2019).

In addition, we categorized the main benefits received and the costs experienced by the local population as mentioned by the respondents during the survey based on the key research question as in Patton (2002) and Ritchie and Spencer (2002). For each type of benefit or cost, the ideas and key points were manually collated to suggest the main point. Furthermore, the level of support as percentages to the BWMA were calculated between the two villages surveyed and across the livelihood strategies of the respondents.

## 3 Results

### 3.1 Respondent Demographic Characteristics

There were nearly equal males and females, within the age groups 18–35 (31%), 36 to 60 (45%), and over 60 years (24%) (Table 1). Most of the household respondents had primary education (54%), secondary education (23%), and postsecondary (6%), but 17% had not attended school.

**TABLE 1:** Respondents' demographic characteristics.

Variable	Category	Number of respondents (*n*)
Village	Minjingu	147
	Vilima Vitatu	155
Gender	Female	152
	Male	150
Age group (years)	18–35	94
	36–60	137
	Above 60	71
Education level	College	18
	Informal	51
	Primary education	165
	Secondary education	68
Length of stay (years)	1–5	8
	6–10	19
	11–15	27
	15 and above	248
Eco‐activities	Agro‐pastoralist	232
	Artisanal weavers	36
	Fish mongers	12
	Small business	22

### 3.2 Perception Toward Benefits of BWMA to the Communities

**FIGURE 2:**
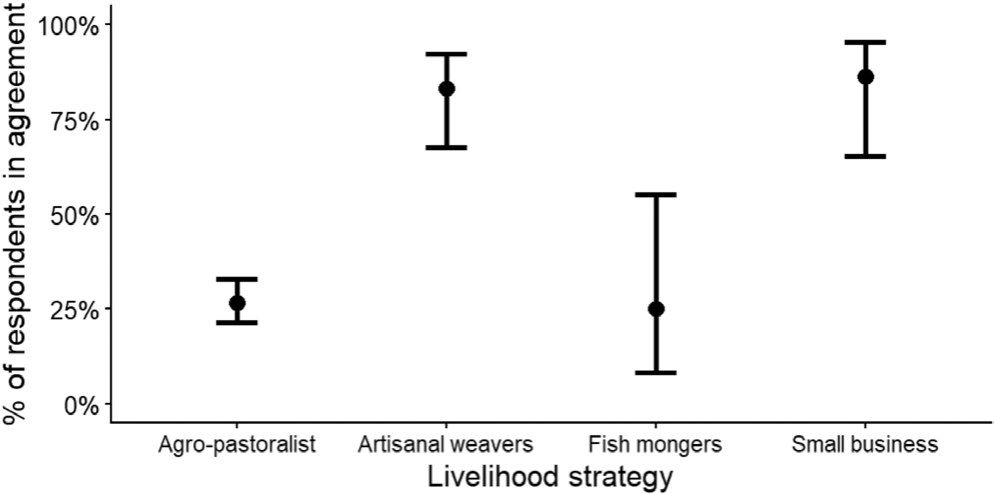
Estimated percentage of respondent agreement on the perception of received BWMA livelihood benefits, based on a binomial multivariate model with respondent livelihood strategy as predictor (error bars at 95% confidence intervals; for details, see Appendix S1).

Overall, most respondents (62%; *N* = 302) reported that they did not benefit from BWMA, with only 38% indicating that they received benefits. At the village level, perceptions did not differ significantly between Minjingu and Vilima Vitatu. However, Vilima Vitatu respondents were approximately 5% more likely than Minjingu respondents to agree that they benefit from BWMA (Vilima Vitatu: 40%, Minjingu: 35%). Furthermore, most of the respondents (73%) from the majority agro‐pastoralist group in the two villages surveyed showed a negative perception toward the benefits received from the BWMA by responding that they do not benefit. In contrast, most of the respondents who deal with small businesses (86%) and weaving activities (83%) responded that BWMA is beneficial to them (Figure 2b), while only a small number of fish mongers (15%) and agro‐pastoralists (27%) acknowledged any benefit. Thus, the livelihood strategy of the respondent was a significant predictor of the perception that BWMA is beneficial to the respondents (*χ* ^2^ = 67.5, df = 3 and *p* < 0.001; Figure 2).

**FIGURE 3:**
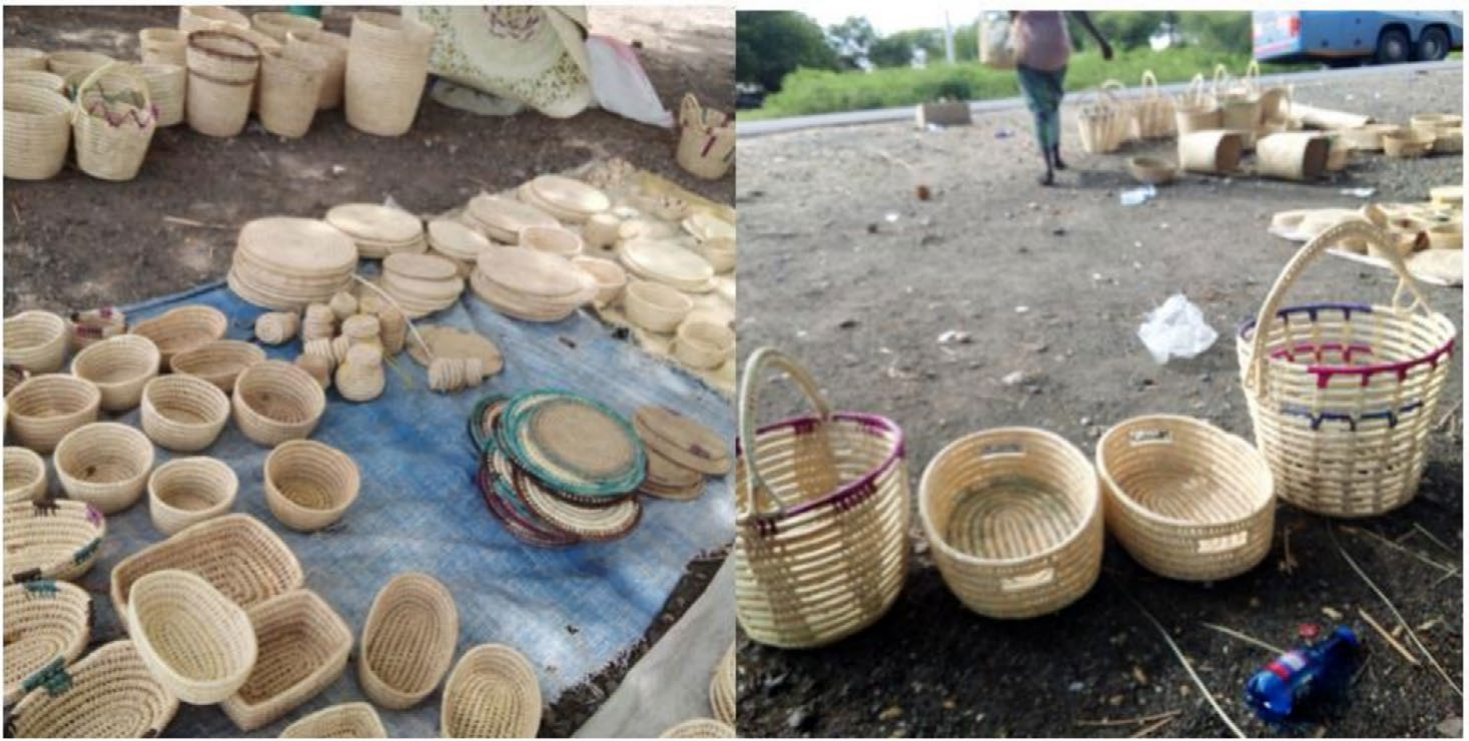
Artisanal weaving items displayed for sale along the Arusha‐Babati Road in Minjingu (Photos taken by Augustino Melembuki during data collection).

The respondents mentioned various benefits they received from BWMA and costs experienced in their main income activity, as summarized in Table 2. For example, agro‐pastoralist respondents mentioned that they are allowed to graze their livestock in designated areas inside BWMA during the dry season, when pasturage in their village land diminishes. Some of the respondents believe that without the BWMA, which protects these areas, the agro‐pastoralists would have settled in them, and there would be no such present option to serve them in times of difficulties such as prolonged drought. In addition, respondents from all livelihood types mentioned that the revenue received by BWMA authority from the central government has helped their villages to construct social service buildings, which include village dispensaries, school classrooms, village offices, and police stations. Similarly, respondents whose livelihood was fishing explained that although fishing activities in Lake Burunge are seasonally closed according to BWMA bylaws, the strategy has increased the number of fish in the lake compared to when they had no such bylaws. Furthermore, respondents whose livelihood was artisanal weaving sold their woven items (Figure 3) to both tourists in transit and locals. They explained that their main weaving materials are protected within BWMA and the harvest is regulated to be sustainable as cited below from one respondent (translated from Kiswahili):

**TABLE 2:** Benefits and costs experienced by the local people with different economic activities around BWMA (*Source:* surveyed responses).

Livelihood activity	Types of benefit received	Type of cost experienced
Agro‐pastoralist	Allowed to graze cattle in designated area inside BWMA during dry season	Increased problematic animals; i.e., crop raiding and livestock depredation
	Construction of; village dispensary, school classrooms, a village office, and a police station	Reduction of village area for grazing and cropping
	Employment in campsites	Increased arrests and harassment by BWMA's Village Game Scouts
	Provided food (grains) during extreme hunger	
	Availability of NTFPs^a^ such as; building poles and firewood	Increased restrictions by BWMA
	Security against dangerous wild animals	Frequent fines to offenders
	Environmental and wildlife protection	Increasing conflicts with protected area authority
Fish mongers	Increasing fish in Lake Burunge	Increased fishing restrictions
	Construction of; village dispensary, school classes, a village office, and a police station	Increased arrests and harassment by BWMA's Village Game Scouts
	Environmental and wildlife protection	
Small business	Increasing business opportunities and sales	
	Construction of; village dispensary, school classes, a village office, and a police station	
Artisanal weavers	Availability and quality of weaving materials in BWMA	Increased arrests and harassment by BWMA's Village Game Scouts
	Construction of; village dispensary, school classes, a village office, and a police station	Increased restrictions to harvest weaving materials

^a^ Non‐timber forest products (NTFPs).

Respondent 1: “We believe that without protection, these materials would have been depleted, as has occurred in many human‐dominated areas.”

### 3.3 Perception Toward Costs of BWMA to the Communities

**FIGURE 4:**
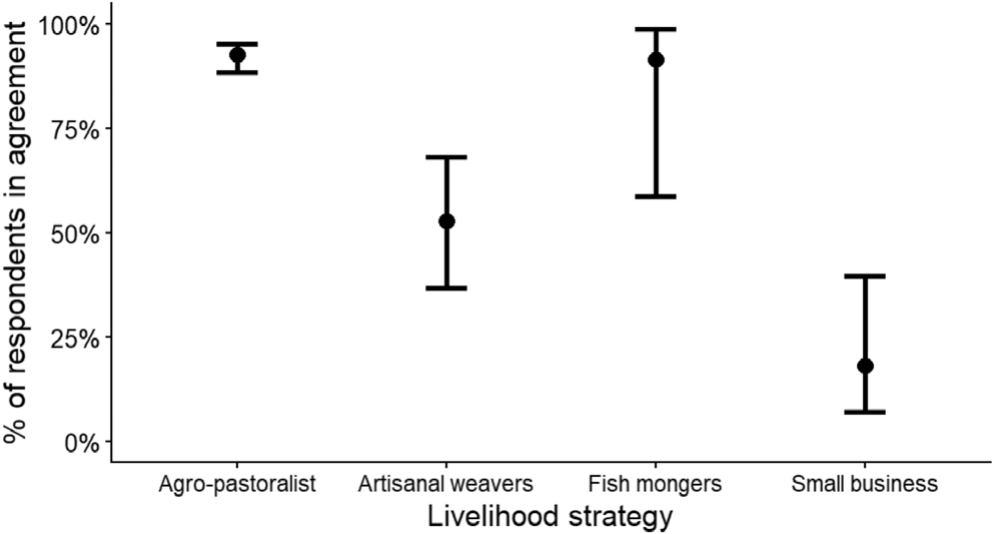
Estimated percentage of respondent agreement on the perception of experienced BWMA livelihood costs, based on a binomial multivariate model with respondent livelihood strategy as predictor (error bars at 95% confidence intervals; for details, see Appendix S2).

Regarding whether local people feel that they experience costs from BWMA to their livelihood activity, most of the respondents (82.5%) agreed with that given statement. Therefore, the respondent livelihood strategy was a significant predictor of the perception that local populations experienced the cost of BWMA (*χ* ^2^ = 81.43, df = 3 and *p* < 0.001; Figure 4). Other factors did not influence the outcome. The most supportive groups were agro‐pastoralists (92.7%) and fishmongers (91.7%), while the least supportive were small business people (18.2%) (Figure 4).

Most respondents identified restrictions imposed by BWMA as a cost to their livelihoods (Table 2). These restrictions were reported to be accompanied by experiences of harassment by Village Game Scouts, fines, and arrests, which in some cases resulted in prosecution and incarceration. Other respondents reported increased interactions with wildlife in their settlements and farms, which they associated with crop damage and livestock losses. Elephants ( *Loxodonta africana* ) and zebras ( *Equus quagga* ) were most frequently mentioned in relation to crop damage, while livestock predation was mainly attributed to spotted hyenas ( *Crocuta crocuta* ) and lions ( *Panthera leo* ).

### 3.4 Perceptions of Support for the Continued Existence of BWMA by the Community

**FIGURE 5:**
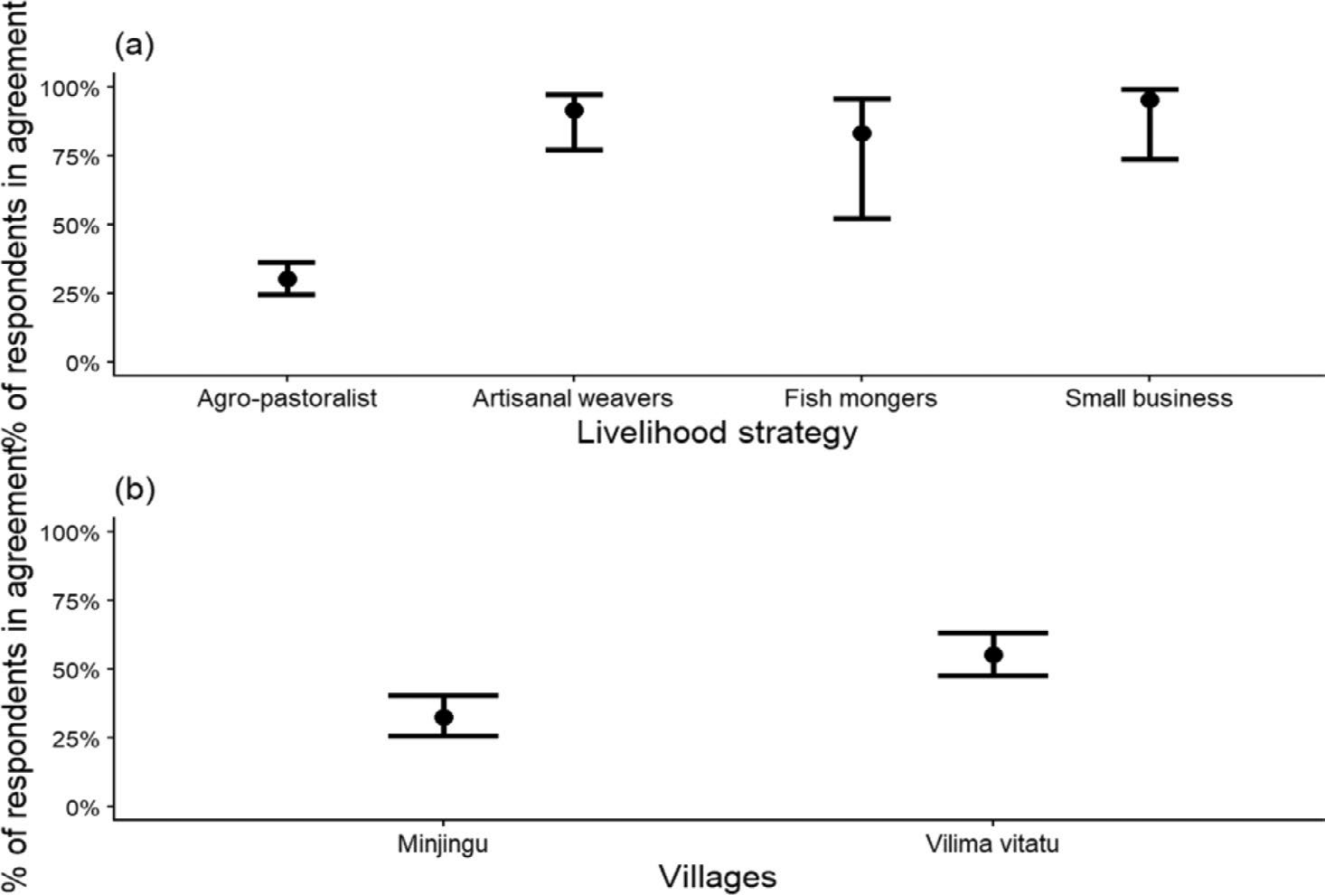
(a, b) Estimated percentage of respondents agreeing on whether they support the existence of BWMA. This is based on a binomial multivariate model with predictors—respondent's livelihood strategy. The error bars are 95% confidence intervals; for details see Appendix S3.

Most of the respondents in Vilima Vitatu (55%) said they currently support the continued existence of BWMA in their area, while only 33% did so in Minjingu village (Figure 5). The respondents' village was a significant predictor of the perception that local people support the continued existence of BWMA (*χ* ^2^ = 16.09, df = 1 and *p* < 0.001; Figure 5b). In terms of their economic activities, most of the respondents whose livelihood activity was fishmonger (83%), small business (95%), and artisanal weaving (92%) said they are currently supporting the continued existence of BWMA in their area (Figure 5). Thus, the economic activities were significant predictors of the perception that local people are supportive of the continued existence of BWMA (*χ* ^2^ = 91.11, df = 3 and *p* < 0.001). However, the livelihood activity of most of the respondents was agro‐pastoralist (70%), and only a small number of them (30%) indicated their support for the continued existence of BWMA.

**FIGURE 6:**
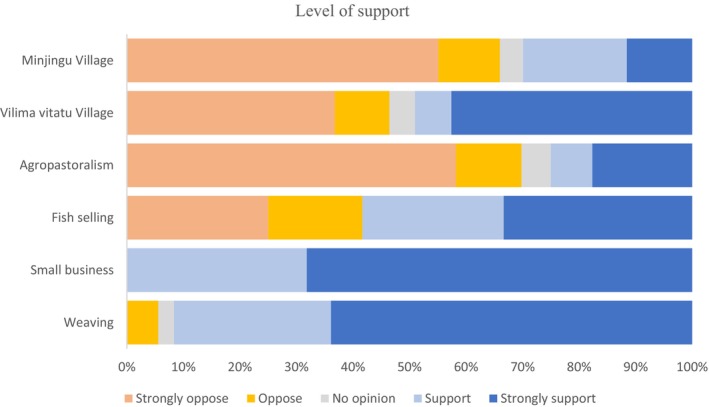
The level of community support for the BWMA by village of residence and by economic activities of the respondents.

To further examine community attitudes, we assessed the intensity of support for BWMA across different villages and livelihood groups (Figure 6). Respondents engaged in small business and artisanal weaving were more likely to strongly support the continued existence of BWMA compared to agro‐pastoralists. In contrast, strong opposition to BWMA was most frequently reported by agro‐pastoralists in both villages. Additionally, respondents who strongly opposed BWMA were more prevalent in Minjingu village than in Vilima Vitatu.

## 4 Discussion

We found that the perception of the local people toward the benefits they received and the costs they experienced from BWMA varied slightly between the two villages surveyed and significantly between the different livelihood strategies practiced in the local communities. In fact, perceptions toward the main benefits and costs of BWMA to communities were directly associated with the livelihood strategies within the respective community group. Similarly, most individuals who supported the continued existence of BWMA in their areas engaged in less common livelihood activities (e.g., fish mongers, small businesses, and artisanal weavers), whereas those who opposed BWMA predominantly relied on agro‐pastoralism. Additionally, a slight majority of those who oppose the continued existence of BWMA are from Minjingu village, probably due to its prolonged conflict with the BWMA.

### 4.1 Perception of the Benefits From and Costs of BWMA

The local perception of the benefits received from BWMA varied between the villages surveyed and local communities' livelihood strategies. At the village level, Minjingu has experienced persistent conflicts, as evidenced by a higher number of unresolved disputes related to benefit sharing and revenue generation with the BWMA than any other village since the establishment of the association over two decades ago (Bluwstein et al. 2016; Moyo et al. 2016; Sulle and Banka 2017; Kicheleri et al. 2018, 2021). The negative perception in Minjingu village started immediately after the establishment of the BWMA. As Sulle and Banka (2017) point out, the village residents claim that they had been generating more revenue before joining BWMA. Similarly, previous studies indicate that Minjingu village had been generating more revenue before joining BWMA and that since then the benefits have decreased markedly due to the BWMA revenue sharing policy which distributes proceeds equally between the participating villages without considering the proportions of revenue generated from individual village lands (Moyo et al. 2016; Sulle and Banka 2017). Because Minjingu village previously generated higher revenues, many respondents believe that, had the village not joined the BWMA, greater investments would have been made in socio‐economic infrastructure such as dispensaries and school classrooms. Furthermore, according to Igoe and Croucher (2007) and Bluwstein et al. (2016), Minjingu village residents claim that there had been insufficient consultation and education before joining the BWMA. In fact, the subsequent amendment of the benefit‐sharing formula regulations (URT 2016a, 2016b) reduced the community share in BWMA in which 5% was allocated to the district councils and 25% to the central government organization (URT 2016a, 2016b; GN 2018, 40). As a result, the villages forming BWMA receive 30% of the total gross revenue collected by the BWMA for development projects (GN 2018, 40; Kegamba, Sangha, Wurm, Kideghesho, and Garnett 2024). These cosmetic reforms of WMAs operations and revenue sharing have entrenched the perception of inequity and continue to demoralize local populations living next to WMAs and serve to exacerbate negative perceptions, which may undermine future conservation goals. In contrast, other community‐based natural resource management (CBNRM) approaches in the Global South, such as the communal conservancies in Namibia and Kenya, allow the communities to retain 100% of the revenues generated from photographic and hunting tourism (Naidoo et al. 2016).

Apart from the conflict over revenue sharing, this study found three other main issues that have allowed negative bias to persist in coloring local perceptions of the BWMA and are compared in the following discussion to other similar CBNRM projects in some of Sub‐Sharan African countries (Table 3): the unresolved conflicts, the ongoing human wildlife conflicts (HWC), and the increasing tensions over access to resources and the restrictions in place.

**TABLE 3:** Specific issues observed in the BWMA which are similar or dissimilar to other CBNRM projects in some sub‐Saharan African countries.

Issues observed in Tanzanian BWMA	Botswana's community development trust	Kenya's conservancies	Namibia's conservancies	Zimbabwean CAMPFIRE	Zambia's ADMADE	References for similarity cases in CBNRM projects
Revenue collection remains centralized, and the community board receive dividend	x	x	x	x	✓	Matenga (2002); Child and Barnes (2010); Tyrrell et al. (2024)
The unresolved land conflicts between BWMA and the nearby communities	x	x	x	x	x	
Human Wildlife Conflicts (HWC) intensified	✓	✓	✓	✓	✓	Gandiwa et al. (2013); Mbaiwa (2014); Gargallo (2021); Chakuya et al. (2025); Kachali and Loos (2025)
Increasing tensions over access to resources and the restrictions in place	✓	✓	✓	✓	✓	Gargallo (2021); Pas et al. (2023); Chitsove and Madebwe (2024); Jani (2025)
The legal right to plan the utilization of natural resources remain centralized, while the community bodies lack real authority	x	x	x	x	✓	Matenga (2002); Child and Barnes (2010); Tyrrell et al. (2024)

*Note:* Key: ✓—similar; x—dissimilar.

The first issue is the long‐standing unresolved conflict between Minjingu village and BWMA over the grazing land. Historical livestock farming practices in East Africa, including Tanzania and therefore local populations near BWMA, are based on an extensive free‐range grazing system within communal areas that has been in place for decades (Butt 2010, 2014). As most of the residents in these two villages are agro‐pastoralists whose grazing land is vital for their livelihood, the conflict over grazing land may therefore influence their perception toward the BWMA.

Second, residents of those villages have been experiencing ongoing human wildlife conflict (HWC) for many years resulting in not only losses to crops, livestock, and property, but also human injuries and sometimes deaths (Tarimo et al. 2021; Raycraft 2023; Hariohay et al. 2024; Kegamba, Sangha, Wurm, Meitamei, et al. 2024). However, several strategies have been implemented by the Tanzanian Government to minimize HWC (Kegamba, Sangha, Wurm, Meitamei, et al. 2024), some of which, especially consolation, have recently been found to be promising in the BWMA and nearby protected areas (Tarimo et al. 2021).

The third issue relates to the increasing tension between the foundational objectives of the BWMA and the realities experienced on the ground. While the BWMA was designed to grant communities greater authority over wildlife management and to enhance conservation and rural livelihoods (Kicheleri et al. 2018), local populations instead face constraints such as restricted access to resources, financial penalties, and potential incarceration. This should not be considered a big challenge because without those restrictions, the WMA will encounter the fate of the vast unprotected areas which contain little if any of the native wildlife. BWMA authority should continue to educate the local people on the importance of having those restrictions for the betterment of wildlife and continued existence of the WMA.

### 4.2 The Perceptions of Minority Livelihood Groups Toward the Benefits of and Costs From BWMA

A small number of respondents from the majority (agro‐pastoralists) explained that the BWMA serves the communities a lot, especially during extreme drought, when they are allowed to supplement the feed in some designated areas. These respondents acknowledged that they would have suffered and lost most of their stocks if they did not have BWMA nearby during the prolonged drought that Tanzania frequently experiences. In the literature, such prolonged drought is frequently associated with climate change, and livestock keepers have been found to struggle in coping with such changes (Lema and Majule 2009; Kideghesho and Msuya 2012). A recent study in the western Serengeti found that most livestock keepers who own large herds tend to illegally supplement their stocks inside Serengeti National Park during the dry season (Matungwa et al. 2024). Our study also found that the minority groups (i.e., those with special livelihood activities near the BWMA) have a similar opinion about it. For example, business owners of shops, restaurants, and guest houses at the village centers were found to have very positive attitudes toward having the BWMA in the area. For them, the BWMA increases the number of tourists (and their guides) in the area, therefore, bringing foreign exchange and creating a multiplier effect for their business growth. Some of them strongly maintained that their village development was highly influenced by tourism activities in the area and that their profits primarily increased during high tourism season, with the opposite holding true during the off season.

Similarly, almost all the respondents whose livelihood strategy involved artisanal weaving were positive about the benefits received from the BWMA. The artisanal weavers obtain their best weaving materials (technically referred to as non‐timber forest products or NTFPs) from BWMA, which they believe would have been depleted if the area had not been protected and their harvest regulated. As Pouliot et al. (2012) noted, NTFP depletion outside protected areas is influenced by several reasons, including overutilization and land degradation. Most of the weavers live in villages bordering the BWMA and the nearby protected areas. They have relatively low incomes and rely on sales of their artisanal weaving (Mugido and Shackleton 2019; Derebe et al. 2023). Income data analysis from similar villages in Bangladesh revealed that poorer households rely more heavily on NTFPs for their livelihood and cash income compared to wealthier households (Kar and Jacobson 2012).

### 4.3 The Level of Support for the Continued Existence of BWMA

This study found a clear division of opinions on the level of support for the continued existence of the BWMA, which is based on their residence and livelihood strategies. Most of the Minjingu village residents are against the continued existence of the BWMA in contrast to those in Vilima Vitatu, which might be associated with the level of existing conflicts between each of these villages and BWMA since its establishment. Our findings align with those of Kideghesho et al. (2007) who found that the level of conflicts between Serengeti National Park and nearby villages was a leading factor that influenced local perceptions toward conservation. Similarly, previous findings in the same villages indicated that Minjingu has more unresolved conflicts with BWMA than any other village and because of this, most of the residents do not support the continued existence of the BWMA (Igoe and Croucher 2007; Bluwstein et al. 2016; Kicheleri et al. 2021). In contrast, there are minority groups of people in the same villages who are very supportive of the existence of the BWMA in their area because it supports their livelihood strategies. BWMA has been well researched in terms of the local perceptions, and its effectiveness in revenue generation is perceived as greater than most of the CBNRM in Tanzania (Moyo et al. 2016; Lee 2018; Kicheleri et al. 2018, 2021; Tang'are and Mwanyoka 2023; Hariohay et al. 2024; Mmbaga et al. 2024). Most of these findings indicated that the residents of Minjingu village do not support the continued existence of this wildlife management area. However, to our understanding, no research has considered the opinions of these groups whose livelihood strategies are in the minority and are highly dependent on the continued existence of the WMA. The voice of these minority groups should be heard and considered.

## 5 Conclusion

This study showed that the perceptions of the respondents toward the benefits received and costs incurred from the existence of the BWMA are influenced by the extent to which those benefits or costs influence the livelihoods of the communities. In addition, the benefits and costs experienced by the communities influenced the level of support by the communities for the continued existence of the BWMA. Thus, the majority of the respondents from the two surveyed villages whose livelihood strategy was agro‐pastoralist showed a negative perception toward the benefits received from the BWMA because, according to them, they would have used the same protected areas as grazing land. Therefore, the majority of the agro‐pastoralists do not support the existence of the BWMA. The main livelihood strategy and the focus for the agro‐pastoralist is raising livestock. This requires sufficient pasturage, which is currently found in large quantity within the BWMA. Outside of it, pasturage has been diminished due to overutilization, lack of land‐use planning, and uncontrolled stocking rates. However, there were small numbers of the livestock keeper respondents who were positive about the benefits received from the BWMA and supported its continued existence. This group acknowledged that the presence of the BWMA in their area acts as a pasture reservoir, especially during prolonged drought, where they were able to obtain special permission to graze in specified zones and therefore preserve their livestock. Similarly, respondents whose livelihood activities were in the minority (such as fishing, small business, and weaving) indicated that they are positive toward the BWMA benefits, as their livelihood strategies are highly dependent on its continued existence. The cost experienced by the local people from the BWMA varied across the livelihood groups. The major costs to the agro‐pastoralists included those related to human wildlife conflict such as crop damage and livestock depredation, while fishers, business people, and weavers expressed their concerns that restrictions imposed on them over access and utilization of resources are the key constraints.

This study recommends that BWMA management adopt more inclusive and participatory approaches that integrate the views of both majority and minority livelihood groups to improve benefit‐sharing and conflict resolution. Special attention should be given to marginalized groups, such as small business owners, fishmongers, and artisanal weavers, whose economic activities depend on BWMA but are often excluded from decision‐making processes. Therefore, investigations of the local perceptions about the management of natural resources should not be limited to those whose livelihood activity places them in the majority. Rather, the voices of those whose livelihood activities are in the minority and to a large extent tend to be ignored should be considered.

Despite those findings, this study has several limitations. First, we surveyed only two purposively selected villages, which may limit the generalizability of the findings to other villages forming the BWMA or other WMAs in Tanzania. Second, data collection relied heavily on self‐reported perceptions, which may be influenced by personal biases, past experiences, and ongoing conflicts, thereby affecting objectivity. Lastly, while the study adopted a mixed‐methods approach, more in‐depth qualitative techniques such as focus group discussions and key informant interviews could provide more insights into local perceptions and conflicts. Future research should therefore expand to a broader geographical scope and incorporate diverse data collection techniques to improve understanding and support evidence‐based conservation planning.

## Author Contributions

**Juma J. Kegamba:** conceptualization (equal), data curation (equal), formal analysis (equal), investigation (equal), methodology (equal), project administration (equal), writing – original draft (equal), writing – review and editing (equal). **Agustino S. Melembuki:** investigation (equal), methodology (equal). **Jackline J. Kyaruzi:** data curation (equal), investigation (equal), methodology (equal).

## Conflicts of Interest

The authors declare no conflicts of interest.

## Supporting information


**Data S1:** Supporting Information.

## Data Availability

The data that support the findings of this study are openly available in “figshare” at https://figshare.com/s/346267dbaa5269e708e9.
