# Supplementary material for: Influence of Livelihood Strategies on Local People Perception Toward the Benefits and Cost of Community‐Based Natural Resource Management: A Case of Burunge Wildlife Management Area, Tanzania
Source: Ecol Evol. 2026 Feb 19;16(2):e73130. doi: 10.1002/ece3.73130 (PMC12920034; doi:10.1002/ece3.73130)
Supplement: Supplementary file 1 — Data S1: Supporting Information. [file ECE3-16-e73130-s001.docx]

**Appendixes**

**Appendix S1**. Probability of agreement of respondent perceptions that they receive benefits from BWMA. These probabilities are based on a binomial multivariable model with village of the respondents and livelihood strategy as predictors. The probabilities shown are averaged across both livelihood strategy. R Least-squares means package (Lenth, 2016) was used to calculate the average estimated probabilities and a confidence interval (CI) of 95% based on the binomial model outputs

| **Livelihood strategy** | **Average probability (%)** | 95% **CI** (%) | |
| --- | --- | --- | --- |
|  |  | **Asymp.LCL** | **Asymp.UCL** |
| Agro-pastoralist | 26.7 | 21.43 | 32.8 |
| Artisanal weavers | 83.3 | 67.54 | 92.3 |
| Fish mongers | 25 | 8.28 | 55.2 |
| Small business | 86.4 | 65.21 | 95.5 |

**Appendix S2**. Respondent’s probability of agreement that they feel they experience costs from BWMA in their livelihood activity. These probabilities are based on a binomial multivariable model with livelihood strategy as predictors. The probabilities shown are averaged across livelihood strategy. R Least-squares means package (Lenth, 2016) was used to calculate the average estimated probabilities and a confidence interval (CI) of 95% based on the binomial model outputs

| **Livelihood strategy** | **Average probability (%)** | **95% CI (%)** | |
| --- | --- | --- | --- |
|  |  | **Asymp.LCL** | **Asymp.UCL** |
| Agro-pastoralist | 92.7 | 88.53 | 95.4 |
| Artisanal weavers | 52.8 | 36.75 | 68.3 |
| Fish mongers | 91.7 | 58.68 | 98.8 |
| Small business | 18.2 | 6.99 | 3.69 |

**Appendix S3**. Respondent’s probability of agreement that they support the continued existence of BWMA. These probabilities are based on a binomial multivariable model with livelihood strategy as predictors. The probabilities shown are averaged across livelihood strategy and the villages of the respondents. R Least-squares means package (Lenth, 2016) was used to calculate the average estimated probabilities and a confidence interval (CI) of 95% based on the binomial model outputs

| **Livelihood strategy** | **Average Probability (%)** | **95% CI (%)** | |
| --- | --- | --- | --- |
|  |  | **Asymp.LCL** | **Asymp.UCL** |
| Agro-pastoralist | 30.2 | 24.6 | 36.4 |
| Artisanal weavers | 91.7 | 77.1 | 97.3 |
| Fish mongers | 83.3 | 52.3 | 95.8 |
| Small business | 95.5 | 73.9 | 99.4 |
| **Village** |  |  |  |
| Minjingu | 32.7 | 25.6 | 40.6 |
| Vilima Vitatu | 55.5 | 47.6 | 63.1 |
